# Effects of Heavy Metal Pollution on the Element Distribution in Hydrobios

**DOI:** 10.3390/toxics12070479

**Published:** 2024-06-30

**Authors:** Chengxin Hu, Chenghao Lai, Ruiyang Yu, Yangfan Chen, Zhixiao Shen, Wei Lu, Xiaofeng Yuan

**Affiliations:** College of Life Science, Zhejiang Chinese Medical University, Hangzhou 310053, China; 202112213902025@zcmu.edu.cn (C.H.); 202112210502022@zcmu.edu.cn (C.L.); 202212211702023@zcmu.edu.cn (R.Y.); 202212211701003@zcmu.edu.cn (Y.C.); 202112210502018@zcmu.edu.cn (Z.S.); 202212211702001@zcmu.edu.cn (W.L.)

**Keywords:** heavy metals, aquatic systems, fish, aquatic plants

## Abstract

At a time when heavy metal pollution is increasing, assessing the levels of contamination and associated health risks is crucial. Samples of water, aquatic plants, and fish were collected from four key areas of heavy metal pollution prevention and control in Zhejiang Province. The levels of elements were analyzed using inductively coupled plasma optical emission spectrometry (ICP-OES). A human health risk model was also developed. The study revealed that heavy metal pollution in the five industrial zones exceeded the national standard for Class V water. Elements like arsenic (As), cadmium (Cd), and chromium (Cr) exceeded permissible levels in aquatic plants across all industrial zones; the exception was lead (Pb). Moreover, the heavy metal concentrations in subject fish tissues collected from each industrial area exceeded safe limits, especially in the gut. According to the human health risk evaluation model, the health risk (1.12 × 10^−3^) and children’s health risk (1.10 × 10^−3^) in these prevention and control zones surpassed the maximum acceptable human risk values. In conclusion, heavy metal elements, along with other pollutants, accumulate and become concentrated in the examined aquatic plants and fish. These pollutants move through the food chain, impacting the entire aquatic ecosystem and posing a health risk to nearby populations.

## 1. Introduction

Zhejiang Province has a reasonably high amount of water resources, which places it in the fourth position in the country consistently throughout the year. Nevertheless, the per capita water resources and use rate in this area are quite low, measuring 375 m^3^/person, which is below the national average and barely a fourth of the global average [1]. In the present situation of diminishing water resources, water pollution has become a major obstacle to sustainable development [2]. In Zhejiang Province, industrial wastewater is the primary cause of pollution. According to statistics, the province has a total sewage discharge of 2.807 billion tons, with industrial wastewater accounting for 66.5% of the total discharge. Industrial wastewater is defined by the presence of heavy metal pollution, which exhibits non-biodegradability, potential toxicity, bioaccumulation ability, and long half-life features. This pollution not only offers a hazard to aquatic organisms but also presents a challenge to humans survival [3,4].

Aquatic species have the ability to accumulate and concentrate heavy metals. When the concentration of heavy metals in the water exceeds the tolerance levels of aquatic plants and animals, it can have a significant toxic impact on various aspects such as immunity, metabolism, and tissues. Heavy metal pollution can lead to a decrease in vitamins, proteins, and other nutrients in plants, ultimately resulting in a reduced crop yields [5,6]. Additionally, the presence of high levels of heavy metal pollution will result in a 50% reduction in the hatching rate of *Pelteobagrus fulvidraco* eggs, as well as an increase in the death rate of young fish by up to 80% [7].

Furthermore, the buildup of heavy metals might potentially interfere with crucial metabolic processes in the human body. Carcinogenic metal ions, such as, have the ability to accelerate redox reactions, resulting in the production of reactive oxygen species (ROS) and components that cause oxidative stress. This process might potentially contribute to the development of many diseases [8,9]. Consuming foods that are contaminated with heavy metals, such as crops and fish, or drinking water contaminated with heavy metals can lead to metabolic irregularities, disruptions in cell genetics, or the development of birth defects or diseases connected to cancer [10,11].

Therefore, we have designated four key regions as focus points for examining heavy metal pollution: Jinhua City, Wenzhou City, Taizhou City, and Jiaxing City. Water, plant, and fish samples were gathered from these regions and analyzed using ICP-OES to ascertain their heavy metal level. This study aims to precisely determine the extent of heavy metal pollution in the water bodies of the designated preventive and control zones in Zhejiang Province by conducting a comprehensive analysis of water pollution. Furthermore, our goal is to assess the overall effect of the distribution of elements in fish and aquatic plants, providing theoretical foundations for water cleanup efforts and accompanying evaluations of health risk.

## 2. Materials and Methods

### 2.1. Samples Collection

**Figure 1:**
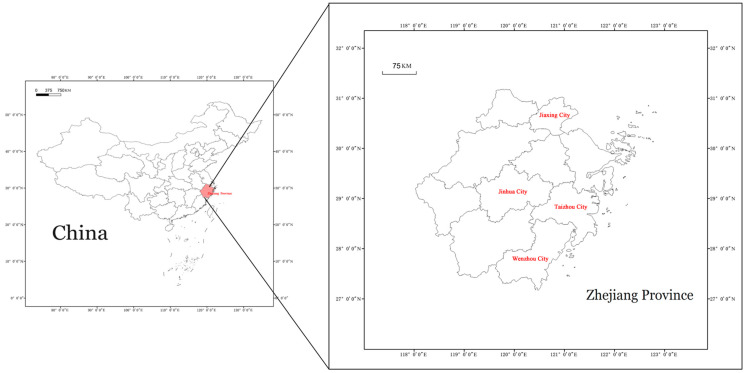
Research area selection map.

Sampling was conducted on polluted river sections within four nationally designated major prevention and control areas (see Figure 1): the Wenzhou Five-Star Industrial Zone (28°03′28.55″ N/120°36′13.29″ E), Jinhua Pujiang Economic Development Zone (29°26′43.30″ N/119°54′48.96″ E), Jiaxing Haining Economic Development Zone (30°44′50.57″ N/120°41′10.40″ E), and Taizhou Huangyan Industrial Zone (28°38′45.74″ N/121°14′24.41″ E). The Nanxi River (28°22.36′42.22″ N/120°45′44.81″ E) was used as a reference control. In the Wenzhou industrial area, samples of *Clarias fuscus* and *Echinochloa phyllopogon* were gathered; in the Jinhua industrial area, samples of *Pistia stratiotes* and *Carassius auratus* were obtained; in the Jiaxing industrial area, samples of *Alternanthera philoxeroides* and *C. auratus* were collected; and in the Taizhou industrial area, samples of *Eichhornia crassipes* and *Hypophthalmichthys molitrix* were collected. The samples were obtained from publicly accessible water bodies where fishing is allowed by the government, with the help of fishermen who have the necessary permissions. Furthermore, the gathering of fish samples and subsequent studies were conducted in strict accordance with ethical rules for the use of animals in scientific research.

In June 2022, we conducted a river near each industrial zone with three sampling points spaced approximately 1 km apart. At each sampling point, one water sample was collected from the surface (0.5 m below the water surface), mid-depth (half depth), and bottom (0.5 m above the riverbed) using polypropylene bottles, which were then thoroughly mixed (each 500 mL, totaling 1500 mL). Three water samples were collected from each industrial zone (totaling 4500 mL), and during the analysis process, each set of three water samples from each industrial zone was divided into 10 subsamples. The water samples were sent to the laboratory, refrigerated, and stored in 1% nitric acid at 4 °C for analysis within 6 h after sampling [12].

Fish are captured using nets or hooks at a rate of 6 per industrial area and promptly transferred to freezers before being transported to the laboratory. When the frozen fish arrives, it is thawed, washed with distilled water, and dissected using a stainless steel knife to remove scales and bones while extracting intestine, muscle, liver, and gill tissue. Any remaining water on the surface is removed with filter paper, and the various tissues are ground. Following mincing, each tissue sample is weighed, dried with a vacuum freeze-drying instrument, crushed into a homogeneous powder, and stored at −20 °C. Aquatic plants are collected by a variety of ways, including mesh bags and digging, and deposited in clean, sealed sample bags [13].

### 2.2. Detection of Physical and Chemical Properties of Water Sample

The process for assessing the physical and chemical characteristics of water samples involved a series of consecutive steps. At first, 100 mL of contaminated water samples was filtered using 25 μm filter papers, and then the filter papers were dried at 60 °C. The desiccated filter papers were subsequently weighed with great care using analytical balances to determine weight variations, which in turn enabled the computation of the water samples’ turbidity index (ss). Additionally, the pH, conductivity (μS/cm), and hardness (ppm) of the filtrate were meticulously measured utilizing a pH meter and a TDS pen, respectively. Moreover, the concentration of phosphate in water sample (mg/L) was determined through the utilization of phosphate reagent, while the ammonia nitrogen content in water was assessed utilizing an ammonia nitrogen rapid detection kit (Hangzhou Lohand Biology Co., Ltd.; Hangzhou, China).

### 2.3. Test of Heavy Metal Content in Polluted Water, Fish, and Plants

We homogenized and mixed water from three heights at each industrial area sampling site, with three replicate groups per industrial area, and then used a disposable syringe to draw 10 mL of the water samples through a 0.45 μm filter for testing. Precisely measuring 0.3 g of plant material, we transferred it into a container along with 7 mL of a solution containing a mixture of HNO_3_ and HClO_4_ in a ratio of 5 to 2. The mixture was subjected to digestion in a controlled environment using a heating furnace at specific temperatures and durations (100 °C for 30 min; 200 °C for 100 min). Upon completion of digestion, the solution was transferred to a fume hood and ventilated to expedite acid evaporation. Once the acid had completely evaporated, ultra-pure water (≥18.0 Ω·cm) was introduced to reach a final volume of 50 mL. Animal samples underwent the same procedures; due to the elevated fat content in fish tissue, the process of digestion was performed twice. The concentration of heavy metal ions in the resulting digestion solution was analyzed using ICP-OES. Each sample was subjected to three treatments to ensure accuracy and reliability.

### 2.4. Setting Up the Human Health Risk Assessment Model

Toxic and harmful pollutants are typically classified based on their cytotoxic and genotoxic properties. Cytotoxicity pertains to substances that are non-carcinogens, whereas genotoxicity encompasses radioactive contaminants and chemical carcinogens [14]. In our study, the presence of radioactive contaminants is so minimal that we can safely ignore them when assessing health risks.

$$\begin{array}{c} R_{j}^{c}=\left[1-ln\left(-D_{j}\times q_{j}\right)\right]/Y\;R_{k}^{n}=\left(D_{k}/RfD_{k}\right)\times 10^{-6}/Y\\ R^{c}=\sum_{j=1}^{j}R_{j}^{c}\;R^{n}=\sum_{k=1}^{k}R_{k}^{n}\;R=R^{c}+R^{n} \end{array}$$

In the formula provided, R^c^ represents the cumulative average individual carcinogenic years caused by various chemical carcinogens present in drinking water, whereas R^n^ denotes the aggregate average individual carcinogenic risks attributed to various cytotoxic substances in drinking water. D signifies the daily exposure dose per unit body mass induced by the chemical carcinogen through the drinking water pathway, whereas RfD_k_ denotes the reference dose of the cytotoxic substance through drinking water. R represents the average individual carcinogenic risk from the cytotoxic substance through drinking water. Standard parameters for adults are calculated based on a weight of 70 kg and a daily drinking water intake of 2 L per person. For children, these parameters are reduced to a weight of 25 kg and a drinking water intake of 1 L per person daily. The average life expectancy is considered 70 years.

### 2.5. Quality and Control

Before analysis, all glassware was washed with distilled water, then immersed in nitric acid (30%) overnight and allowed to air-dry. Each batch of samples was blanked, and all samples were analyzed simultaneously to ensure the experiments’ precision. Standard samples were rechecked after every 10 samples analyzed. The recovery rates for specific metals using the analytical methods ranged from 75% to 109%. All acids used in digestion were of standard analytical grade. Contaminants did not impact the analysis, and the relative standard deviation (RSD) for all tests was ≤10% [15].

### 2.6. Statistics and Analysis

The data obtained were analyzed using SPSS 19.0 software. The data were presented using appropriate statistical formulas. Correlation analyses were conducted to investigate the relationships between the physical and chemical properties of water, as well as the elemental contents in water, animal, and plant samples.

## 3. Results

### 3.1. Physical and Chemical Properties of Water Samples in Industrial Areas

The pH levels of the rivers in each industrial area are documented in Table 1. Notably, the pH levels of the water in the Jinhua, Jiaxing, and Taizhou industrial areas were significantly lower than that of the Nanxi River water. Furthermore, the industrial areas exhibit higher levels of hardness, turbidity, ammonia nitrogen content, total phosphorus (TP), and electrical conductivity compared to uncontaminated water, indicating significant differences. This demonstrates the contamination of river waters surrounding these industrial zones.

### 3.2. The Metal Content of Water Sample in Each Industrial Area

The metal element concentrations of water samples from each industrial area are shown in Table 2. A comparison with the “People’s Republic of China Surface Water Environmental Quality Standard” V-type surface water standard reveals that the heavy metal concentrations of all industrial areas exceed regulatory thresholds, with the exception of Mn. Water sources in these industrial areas have been found to be polluted with heavy metals to varying degrees. Notably, the contents of As in all heavy metal polluted industrial areas exceeded the standard by more than 86 times; Cd exceeded the standard by more than 20 times; Cr exceeded the standard by more than 6 times; Cu exceeded the standard by more than 154 times; Fe exceeded the standard by more than 24 times; Pb exceeded the standard by more than 110 times; Zn exceeded the standard by more than 50 times. Mn exceeded the standard by more than 2 times. In conclusion, the heavy metals in these four industrial zones have exceeded the standard, but the severity of heavy metal pollution varies by location, which is closely related to the type of industrial zones.

### 3.3. The Contents of Heavy Metals in Aquatic Plants

Table 3 shows the metal element content of plants in the water samples from each industrial area. Plants in industrial water samples exceed national standards to varying degrees. Mn, Zn, and Fe in *E. phyllopogon* in Wenzhou industrial area exceeded the standard, of which Mn exceeded the standard by 1.4 times, Zn exceeded the standard by 1.2 times, and Fe exceeded the standard by 2.7 times. As, Cd, Cr, Mn, Zn, Fe, and Al in *P. stratiotes* in the Jinhua area exceeded the standard, and Cu and Pb did not exceed the standard. Cd, Cr, and Fe in *A. philoxeroides* in the Jiaxing area exceeded the standard, and As, Cu, Mn, Pb, Zn, and Al did not exceed the standard. For *E. crassipes* in the Taizhou area, Cd, Cr, Cu, Mn, Zn, Fe, and Al exceeded the standard, and As and Pb did not exceed the standard. It is clear that the heavy metal elements in the plants in the water samples of each industrial area have varying degrees of exceedance, indicating that heavy metal pollution in water will be transmitted to plants, with the exception of Pb.

### 3.4. The Content of Heavy Metal Elements in Fish

The national standard for food safety (GB2762-2012) [16] states that the heavy metal content of edible fish must meet the following conditions: Pb ≤ 0.5 mg/kg; Cd ≤ 0.05 mg/kg; As ≤ 0.5 mg/kg; and Cr ≤ 1.0 mg/kg. A comparison revealed (as shown in Table 4) that heavy metals in fish muscle and viscera in effluents from the various industrial districts exceeded the standard and did not meet the edible standard. Among them, the muscle of *C*. *fuscus* in Wenzhou Industrial Zone exceeded the standard of Cr by 2.7 times and Pb exceeded the standard by 1.4 times; Cd was not more than the standard. *C*. *auratus* from Jinhua Industrial Zone, like *H*. *molitrix* from Taizhou Industrial Zone, exceeded muscle standards for As, Cr, Pb, and Zn while also exceeding Cd and Cu standards. *C*. *auratus* from Jiaxing Industrial Zone had excesses of As, Pb, Cr, and Zn but no excesses of Cd.

Moreover, the comparison of heavy metal content in fish from four different locations revealed that *C. fuscus*, *H. molitrix*, and *C. auratus* have higher intestinal heavy metal content than other parts, owing to food residue accumulation in the intestines. This finding suggests that heavy metals from plants accumulate in the intestines via the food chain, eventually affecting animals. Additionally, Pb and Zn accumulate in the gills of *C. fuscus*, *H. molitrix*, *or C. auratus*, with higher levels in the gills compared to the liver and muscle. Meanwhile, Cu accumulation is notably pronounced in the liver. Heavy metal concentrations in fish muscle are generally lower than in other parts, indicating that heavy metals accumulate more in viscera than in muscle tissue. Even so, heavy metal levels in fish muscle remain excessive and unfit for consumption, highlighting the negative impact of heavy metal pollution on fish growth.

### 3.5. Analysis of Elements in Aquatic Plants in Water Sample

**Figure 2:**
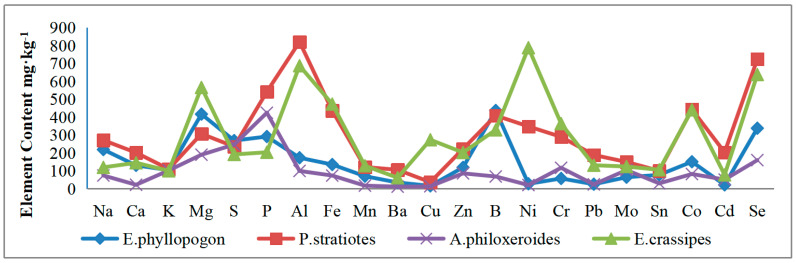
Distribution of elements in aquatic plants in wastewater.

The uptake of elements essential for aquatic plants primarily occurs from water and sludge, so water contamination has an unavoidable impact on plant growth. Utilizing ICP-OES, the content of twenty-two elements in aquatic plants was determined and their distribution was plotted (see Figure 2). To improve clarity, the content of certain elements was magnified several times: B, Cr, Ni, and Pb by 10 times; Mo, Sn, Cd, and Co by 100 times; Se by 1000 times. Conversely, the content of Al, Fe, Mg, Mn, P, and S was reduced by 10 times and Na, Ca, and K by 100 times. Analysis of the element distribution in aquatic plants reveals consistency in the contents of *P. stratiotes* (JH) and *E. crassipes* (TZ), whereas *E*. *phyllopogon* (WZ) and *A. philoxeroides* (JX) exhibit similar trends. *P. stratiotes* and *E. crassipes* are floating plants, whereas *E. phyllopogon* and *A. philoxeroides* are emergent aquatic plants. It is evident that the absorption patterns of elements by floating and emergent plants correspond to their respective categories. Moreover, floating and emergent plants exhibit distinct element absorption patterns.

Furthermore, floating water plants like *P. stratiotes* and *E. crassipes* have greater ability to absorb heavy metals than emergent plants like *E. phyllopogon* and *A. philoxeroides*. Floating water plants are particularly effective at absorbing heavy metals such as Cu, Ni, Co, Cr, and Pb, with strengths 3–20 times greater than emergent plants in this regard.

For showing all the elements in a clear figure, we amplify the contents of B, Cr, Ni, Pb 10 times; Mo, Sn, Cd, Co 100 times; and Se 1000 times and reduce the contents of Al, Fe, Mg, Mn, P, S 10 times and Na, Ca, K 100 times. 

### 3.6. Analysis of Element Content in Fish in Water Sample

Fish, as every knows, have the ability to absorb and accumulate waterborne elements as they move up the food chain. The distribution of 22 elements across various tissues and organs, including gills, muscles, liver, and intestine, was analyzed using ICP-OES, as illustrated in Table 5. In order to distinguish between the two different areas of *C. auratus*, H for Jinhua and X for Jiaxing are used after the corresponding legend.

The results showed a significant accumulation of heavy metals in the intestines of *C. fuscus* and *H. molitrix*, which outperformed other samples. The concentrations of Pb, Cr, Cd, and Ni in the intestines of *C. fuscus* were markedly higher than in other samples. Additionally, Mn content in the intestine of *H. molitrix* increased significantly above normal levels. The intestines have the greatest capacity for heavy metal enrichment, followed by the gills, with the lowest levels found in the muscle tissue. However, it is noteworthy that heavy metal concentrations in all organs exceed the standard limits. These findings underscore the unequivocal impact of heavy metal pollution on fish. 

### 3.7. Human Health Risk Assessment Model

According to the human health risk assessment model that takes heavy metal contents into account, the calculated health risk for adults in these control areas is 1.12 × 10^−3^, while for children, it is 1.1 × 10^−3^. Both values exceed the maximum acceptable risk to human health, which is 1 × 10^−4^ (Table 6). This implies that heavy metal pollution is likely to contaminate the drinking water of nearby residents, substantially elevating the risk of carcinogenesis among the general population.

### 3.8. Analysis of the Correlation between Heavy Metal Elements in Wastewater and Fish

The correlation analysis reveals strong links between heavy metal elements in water and those in fish. A significant negative correlation exists between Cr levels in water and those in *C. fuscus* and *H. molitrix* from the industrial areas of Wenzhou and Taizhou. Additionally, a significant negative correlation exists between As content in river water and that in *C. auratus*. As a result, it can be concluded that Cr and As concentrations in water are significantly negatively correlated with those in fish, whereas no significant correlation was found between heavy metals and plants.

## 4. Discussion

Our study underscores the ongoing presence of heavy metal pollution in National Heavy Metal Pollution Control Areas such as Jinhua, Jiaxing, Wenzhou, and Taizhou. The water quality in these areas is typically below the Type V surface water standards, making it unfit for drinking and even irrigation. As a consequence of water pollution, plants and fish exhibit varying degrees of adverse effects, including mortality or impaired growth. Such detrimental impacts extend to the surrounding ecological environment, amplifying the risk of cancer among nearby residents.

In general, heavy metals enter fish bodies via three main pathways: gills, the alimentary canal, and the skin [17]. Our experimental results showed that heavy metal concentrations were highest in the intestine, followed by the gills and liver, and lowest in the muscle, which was consistent with previous studies [18]. This accumulation in the intestine is caused by fish’s ability to accumulate heavy metals through ingestion, where unabsorbed heavy metals accumulate in the digestive tract, resulting in higher concentrations [19]. Heavy metals cannot be decomposed within fish, so they accumulate in the liver over time, resulting in heightened concentrations. Additionally, gills play crucial roles in respiration, osmoregulation, excretion, and pH regulation, serving as primary conduits for metal ion exchange in water and having a large surface area that allows for rapid diffusion of metal ions [20,21,22]. Fish muscles typically have lower heavy metal content compared to other body parts, indicating a higher likelihood of heavy metal enrichment in viscera than muscles [23,24].

*H. molitrix*, *C. auratus*, and *C. fuscus* have distinct feeding habits: *H. molitrix* filter-feeds, *C. auratus* is an omnivorous bottom-dweller, and *C. fuscus* is a carnivorous bottom-dweller. Previous studies have suggested that omnivorous and herbivorous fish may accumulate higher levels of heavy metals due to their feeding habits and associations with heavy metals [25]. For example, metal levels such as Cr and Co in *H. molitrix* were found to be higher than in *C. auratus*, suggesting a simpler food chain for *C. auratus* in certain environments. In our study, Cu and Zn enrichment in liver was significantly higher than that in gill and muscle, and Cu accumulation in liver was even higher than that in intestine in Wenzhou *H. molitrix*. However, unlike Salami IR [26], we did not see the enrichment of Pb in liver or gills. This suggests that the different accumulation patterns of heavy metals in fish tissues may be closely related to the different feeding habits and living environments of different fish species and that the removal and transport of heavy metals may lead to different distributions of heavy metals in various tissues, or it may be due to the fact that Pb itself does not exceed the standards in the river water [27]. Furthermore, Cr and As in water showed significant negative correlations with fish species, which were found to be biologically declining in freshwater food webs [28]. Our study revealed that *C. fuscus* had significantly higher Cr content than herbivorous fish, possibly due to their higher trophic level that converts it into forms that are resistant to biological reduction in freshwater ecosystems [29]. Jiaxing has significantly higher levels of As, Cr, and Pb in the intestines of *C. auratus* compared to Jinhua, while Jinhua has higher levels of Cu and Zn in the liver and gills of the fish. Heavy metal accumulation varies among *C. auratus* samples from Jiaxing and Jinhua. The reason for this could be that when the benthic animal *C. auratus* feeds on benthic animals and submerged plants, not only the heavy metal content of the water but also the influence of sediments must be taken into account [30,31]. There is no obvious correlation between heavy metal elements in water and internal elements in fish, except for As and Cr. Water quality may not be the main factor affecting metal concentration in fish [32].

Heavy metal pollution has a significant impact on animal life and plant growth. Heavy metals possess the capability to impede seed germination, disrupt the functionality and efficiency of the photosynthetic system, and inhibit stomatal function and cambium activity, consequently affecting overall plant productivity [5,33]. Metal accumulation in aquatic plants varies by anatomical part [34], and metal accumulation within organisms fluctuates in response to changes in water pH [35]. A high concentration of Zn and Cd was detected in *P. stratiotes*, possibly due to the high conductivity of Jinhua’s water, which allows for high cation mobility [36]. *A. philoxeroides* is resistant to toxic metal exposure [37], but in this study, the potential to stabilize Pb or Zn as seen in Sudarshan P [38] was not observed. There has been little research into heavy metal accumulation in the related *E. phyllopogon*, which may unsuitable for water body restoration due to its low absorption of heavy metals. Considering that *E. crassipes* is a large metal-accumulating plant and considered a sensitive species [39], there have been many related studies on water hyacinth for water body restoration [40,41], and the accumulation of heavy metals in this study has reached high levels. Currently, there is no significant correlation between heavy metals in water and plants. The high concentration of heavy metals in aquatic plants suggests that certain species may accumulate high levels of metals even if the metal concentration in the water is not particularly high [42].

*E*. *crassip*, *E*. *phyllopogon*, and *P. stratiotes* had the highest Mn enrichment rates, surpassing 1000-fold, suggesting their potential as hyper-accumulators of Mn. Moreover, *E*. *crassip* and *P. stratiotes*, floating plants, are more effectively enriched with heavy metals than *E. phyllopogon* and *A. philoxeroides*, submersed plants. This disparity may stem from the floating plants primarily absorbing heavy metal ions [43], which are dissolved in water, from their roots. These roots are rich in cellulose [44], which can promote heavy metals to adsorb into them [45]. At the same time, emergent aquatic plants are rooted in the sediment, absorbing nutrients primarily from the deep soil. Consequently, heavy metals tend to concentrate in the surface sediment [46]. Additionally, the low oxygen content in deep soil makes it easy for heavy metal elements to combine with volatile sulfides to form insoluble precipitates [47]. This process slows down the rate at which emergent aquatic plants’ are enriched with heavy metals.

In this study, we assessed the cancer risk for adult inhabitants and children living nearby. The findings reveal that the risks of developing cancer for both adults and children exceed the maximum acceptable threshold. As a result, we advocate for the continuous periodic monitoring of heavy metal concentrations in the rivers of these industrial areas. Furthermore, to protect public health, aquatic organisms, particularly edible species, must be closely monitored. 

Investigating the presence and accumulation of heavy metal elements and other pollutants in different industrial zones can be used to directly determine the causes of characteristic changes in certain aquatic species, as well as ecological risks [48]. We have a limited understanding of the mechanisms and pathways of heavy metal ion absorption and accumulation in aquatic organisms, as well as consideration of the cumulative, synergistic, or antagonistic toxic effects of specific heavy metals [49]. Further research should look into the impacts of heavy metal accumulation on various growth stages of aquatic organisms, exposure time, and external environmental changes (such as sediment).

## Figures and Tables

**Table 1:** Physical and chemical properties of wastewater from key heavy metal pollution prevention and control areas in Zhejiang Province ($\bar{x}\pm s$, n = 30).

	pH	Hardness ppm	Turbidity g/L	Ammonia Nitrogen mg/L	Phosphorus mg/L	Conductivity μS/cm	Color
Wenzhou Industrial Zone	7.5 ± 0.2	126.9 ± 19.9 *	0.3 ± 0.1 *	9.5 ± 1.1 **	0.3 ± 0.1 **	274.5 ± 47.6 *	red
Jinhua Industrial Zone	6.7 ± 0.3 **	196.4 ± 34.3 **	0.6 ± 0.2 **	0.7 ± 0.2	0.2 ± 0.1 *	355.7 ± 48.2 **	yellow
Jiaxing Industrial Zone	7.2 ± 0.5 **	170.2 ± 20.8 **	0.3 ± 0.1 *	0.1 ± 0.0	0.1 ± 0.0	357.6 ± 26.5 **	yellow
Taizhou Industrial Zone	7.2 ± 0.5 **	90.6 ± 23.7	0.6 ± 0.1 **	1.1 ± 0.2	0.2 ± 0.1 *	264.8 ± 32.9*	yellow
Comparison of Nanxi River Water	7.8 ± 0.2	10.5 ± 0.5	0.1 ± 0.0	0.0 ± 0.0	0.0 ± 0.0	44 ± 5.2	-

* indicates a significant difference in the indicator compared to the control group (*p* < 0.05) and ** indicates a very significant difference (*p* < 0.01).

**Table 2:** Metal and elemental content in wastewater from key heavy metal pollution prevention and control areas in Zhejiang Province (mg/L) ($\bar{x}\pm s$, n = 30).

	As	Cd	Cr	Cu	Fe	Al	Pb	Zn	Mn
Wenzhou Industrial Zone	5.1 ± 0.7 **	0.1 ± 0.1	0.3 ± 0.1	7.6 ± 0.3 **	14.6 ± 7.3	13.1 ± 2.7	2.9 ± 0.6 **	23.8 ± 7.2 **	241.3 ± 29.5 **
Jinhua Industrial Zone	7.8 ± 1.8 **	0.1 ± 0.1	0.5 ± 0.2	8.9 ± 0.4 **	11.5 ± 5.7	1.1 ± 0.6	2.4 ± 0.7 **	3.5 ± 0.5	0.3 ± 0.1
Jiaxing Industrial Zone	4.3 ± 1.2 **	0.1 ± 0.1	0.5 ± 0.2	3.7 ± 0.4	7.3 ± 2.2	1.3 ± 0.4	1.1 ± 0.4 **	2.5 ± 0.6	0.2 ± 0.1
Taizhou Industrial Zone	6.3 ± 1.5 **	0.4 ± 0.1 **	19.3 ± 4.1 **	7.7 ± 0.7 **	11.7 ± 4.8	24.1 ± 18.0	1.6 ± 0.2 **	18.7 ± 5.1 **	0.6 ± 0.2
Class V water standard	0.05	0.005	0.05	0.02	0.3	-	0.01	0.05	0.1

** denotes a highly significant difference (*p* < 0.01).

**Table 3:** Heavy metals and other elements in aquatic plants in wastewater from key prevention and control areas of heavy metal pollution in Zhejiang Province (mg/kg) ($\bar{x}\pm s$, n = 30).

	As	Cd	Cr	Cu	Mn	Pb	Zn	Fe	Al
Wenzhou Industrial Zone *E. phyllopogon*	1.4 ± 0.1 **	0.3 ± 0.1	5.7 ± 0.2	16.2 ± 0.6 **	705 ± 8.0 **	2.5 ± 0.4 **	119.9 ± 9.2 **	1360 ± 123.0 **	1742 ± 41.1 **
Jinhua Industrial Zone *P. stratiotes*	10.5 ± 0.4 **	2.0 ± 0.2 **	28.9 ± 0.6 **	36.8 ± 1.4 **	1212 ± 18.1 **	18.7 ± 0.8 *	222.6 ± 1.5 **	4362 ± 104.7 **	8206 ± 406.3 **
Jiaxing Industrial Zone *A. philoxeroides*	0.9 ± 0.1 **	0.5 ± 0.3	11.7 ± 0.2	14.0 ± 0.5 **	174.6 ± 2.4 **	2.5 ± 0.9 **	86.5 ± 1.1 **	741.6 ± 41.0 **	1001.9 ± 17.9 **
Taizhou Industrial Zone *E. crassipes*	4.0 ± 0.1 **	0.8 ± 0.1 **	36.4 ± 4.2 **	274 ± 3 **	1282 ± 3.1 **	13.1 ± 0.3 **	202.4 ± 0.1 **	4730 ± 76.3 **	6871 ± 29.0 **
National standard	5.0	0.3	10.0	50.0	500.0	20.0	100.0	500.0	3000.0

* denotes a significant difference from the national standard (*p* < 0.05), ** denotes a highly significant difference (*p* < 0.01).

**Table 4:** Heavy metal element content in fish from wastewater in key prevention and control areas of heavy metal pollution in Zhejiang Province (mg/kg) ($\bar{x}\pm s$, n = 30).

		As	Cd	Cr	Pb	Cu	Zn
Wenzhou Industrial Zone *C. fuscus*	liver	0.6 ± 0.1	0.1 ± 0.0	3.3 ± 2.3	0.9 ± 0.2	40.3 ± 7.3 *	133.9 ± 28.9
	gill	1.0 ± 0.6 **	0.1 ± 0.0	2.0 ± 0.2	1.2 ± 0.2	4.1 ± 1.5 **	86.2 ± 3.1
	muscle	0.4 ± 0.2	0.0 ± 0.0 **	2.7 ± 0.6	0.7 ± 0.2	1.6 ± 0.1 **	41.6 ± 5.4 *
	intestines	7.1 ± 0.8 **	3.3 ± 0.2 **	200.6 ± 56.8 **	41.7 ± 6.4 **	142.7 ± 4.5 **	573.5 ± 70.7 **
Jinhua Industrial Zone *C. auratus*	liver	0.8 ± 0.0 *	0.2 ± 0.0	1.1 ± 0.1	0.8 ± 0.1	14.7 ± 0.3 **	310.1 ± 0.2 **
	gill	0.6 ± 0.2	0.1 ± 0.0	0.4 ± 0.1	1.0 ± 0.0	4.6 ± 0.8 **	364.1 ± 12.9 **
	muscle	0.7 ± 0.1 *	0.0 ± 0.0 **	1.9 ± 0.0	0.6 ± 0.0	2.3 ± 0.2 **	91.5 ± 0.5
	intestines	1.6 ± 0.0 **	0.5 ± 0.0 **	4.0 ± 0.1	3.5 ± 0.6 *	17.6 ± 0.3 **	295.3 ± 14.5 **
Jiaxing Industrial Zone *C. auratus*	liver	0.4 ± 0.2 *	0.1 ± 0.0	1.4 ± 0.1	0.8 ± 0.1	8.8 ± 0.1 **	188.9 ± 16.8 **
	gill	0.7 ± 0.1 *	0.0 ± 0.0 **	2.9 ± 0.9 *	0.8 ± 0.0	3.1 ± 0.0 **	218.9 ± 1.6 **
	muscle	0.5 ± 0.0	0.0 ± 0.0 **	2.1 ± 0.1	0.6 ± 0.0	2.5 ± 0.0 **	101.8 ± 4.9
	intestines	5.1 ± 0.3	0.9 ± 0.0 **	41.7 ± 5.7 **	22.6 ± 6.8 **	33.9 ± 2.2	280.0 ± 7.7 **
Taizhou Industrial Zone *H. molitrix*	liver	0.8 ± 0.2 *	0.1 ± 0.0	0.9 ± 0.0	0.9 ± 0.2	60.2 ± 2.1 **	120.0 ± 4.6 *
	gill	0.7 ± 0.1 *	0.0 ± 0.0 **	0.5 ± 0.0	1.1 ± 0.3	3.1 ± 0.1 **	139.4 ± 10.1 **
	muscle	0.7 ± 0.1 *	0.0 ± 0.0 **	1.9 ± 0.1	1.0 ± 0.2	2.2 ± 0.0 **	34.6 ± 1.9 **
	intestines	5.5 ± 0.3	1.0 ± 0.0 **	19.6 ± 0.6 **	10.0 ± 0.3 **	32.7 ± 2.0	152.3 ± 9.0 **
National standard		0.5	0.05	1.0	0.5	30	100

* denotes a significant difference from the national standard (*p* < 0.05), ** denotes a highly significant difference (*p* < 0.01).

**Table 5:** Distribution of elements in fish bodies (mg/kg) (n = 30).

	Fe	As	Mn	Ba	Cu	Zn	Ni	Cr	Pb	Mo	Sn	Co	Cd	Se
*C. fuscus* liver	601.85	79.93	84.08	2.679	33.739	111.957	0.645	1.163	0.69	3.182	1.691	0.245	0.103	9.255
*C. auratus* liver H	472.48	65.36	143.88	14.896	14.675	310.092	0.426	1.062	0.832	0.455	0.467	0.213	0.176	1.753
*C. auratus* liver X	656.22	127.59	73.74	4.739	8.771	188.912	2.311	1.437	0.815	0.497	0.632	0.242	0.083	4.084
*H. molitrix* liver	577.92	145.99	167.59	2.922	60.173	119.956	0.332	0.954	0.927	1.365	0.683	0.231	0.053	4.048
*C. fuscus* gill	422.44	48.83	125.95	3.493	5.447	88.126	0.994	2.067	1.057	0.222	0.655	0.352	0.075	2.362
*C. auratus* gill H	215.15	71.90	282.61	54.827	4.562	364.052	0.135	0.367	1.038	0.135	0.464	0.057	0.049	1.227
*C. auratus* gill X	393.88	111.01	250.82	27.726	3.053	218.89	0.465	2.895	0.819	0.145	0.457	0.103	0.029	1.885
*H. molitrix* gill	295.07	90.16	670.43	38.422	3.083	139.451	0.194	0.503	1.103	0.091	0.651	0.177	0.021	1.726
*C. fuscus* flesh	196.85	84.58	55.09	2.453	1.732	37.72	0.596	2.661	0.604	0.074	0.951	0.103	0.021	2.662
*C. auratus* flesh H	162.31	119.23	60.56	8.913	2.344	91.543	0.164	1.921	0.603	0.082	0.521	0.025	0.033	0.731
*C. auratus* flesh X	184.37	122.27	77.14	6.486	2.464	101.85	0.387	2.061	0.616	0.102	0.497	0.045	0.016	2.363
*H. molitrix* flesh	144.48	196.89	79.79	4.006	2.151	34.617	0.219	1.865	1.027	0.049	0.713	0.028	0.012	1.389
*C. fuscus* intestine	10761.21	131.11	2073.74	147.695	145.213	637.483	98.226	251.334	47.442	6.121	2.271	13.311	3.428	2.648
*C. auratus* intestine H	1599.2	129.38	980.68	33.699	17.569	295.362	1.036	4.019	3.461	0.588	0.505	0.567	0.493	1.080
*C. auratus* intestine X	184.37	120.91	77.14	6.486	2.464	101.85	0.387	2.061	0.616	0.102	0.497	0.045	0.016	2.363
*H. molitrix* intestine	9664.39	42.02	2725.96	105.576	32.687	152.244	9.647	19.611	9.963	0.589	0.367	4.432	0.952	1.089

**Table 6:** Human health risk evaluation model R-values.

	Carcinogenic Heavy Metals			Figure	Non-Carcinogenic Heavy Metals			Figure	Total Health Risk	Maximum Acceptable Risk
	Cr	As	Cd		Cu	Zn	Pb			
adults	3.55 × 10^−4^	2.79 × 10^−4^	4.89 × 10^−4^	1.12 × 10^−3^	1.69 × 10^−12^	8.86 × 10^−14^	2.32 × 10^−12^	4.11 × 10^−12^	1.12 × 10^−3^	1 × 10^−4^
children	3.49 × 10^−4^	2.66 × 10^−4^	4.82 × 10^−4^	1.10 × 10^−3^	2.37 × 10^−12^	1.24 × 10^−13^	3.25 × 10^−12^	5.75 × 10^−12^	1.10 × 10^−3^	1 × 10^−4^

## Data Availability

The data presented in this study are available on request from the corresponding author. The data are not publicly available due to the requirements of the research institution.

## References

[1] Tian X.Z., Wang B.Q., He X.J., Xia D.M. (2021). Analysis and prediction of water resources development and utilization in Zhejiang Province. Zhejiang Hydrotech..

[2] Wang T., Zhang J., Li Y., Xu X., Li Y., Zeng X., Huang G., Lin P. (2022). Optimal design of two-dimensional water trading based on risk aversion for sustainable development of Daguhe watershed, China. J. Environ. Manag..

[3] Emenike E.C., Iwuozor K.O., Anidiobi S.U. (2022). Heavy Metal Pollution in Aquaculture: Sources, Impacts and Mitigation Techniques. Biol. Trace Elem. Res..

[4] Oyugi A.M., Kibet J.K., Adongo J.O. (2021). A review of the health implications of heavy metals and pesticide residues on khat users. Bull. Natl. Res. Cent..

[5] Pokorska-Niewiada K., Rajkowska-Myśliwiec M., Protasowicki M. (2018). Acute Lethal Toxicity of Heavy Metals to the Seeds of Plants of High Importance to Humans. Bull. Environ. Contam. Toxicol..

[6] Zhu J.L., Xu W.J., Guo S.C., Zhou J.Y., Lu T., Xing P.H., Cai Q., Sun R. (2022). Harm of heavy metal pollution in water body and its control technology. Mod. Agric. Sci. Technol..

[7] Bo S.J., Xu Z.R. (2006). Effects of cadmium on mitochondrial structure and energy metabolism in gill of Pelteobagrus fulvidraco. Acta Appl. Ecol..

[8] Adetutu A., Adegbola P.I., Aborisade A.B. (2023). Heavy metal concentrations in four fish species from the Lagos lagoon and their human health implications. Heliyon.

[9] Fu Z., Xi S. (2020). The effects of heavy metals on human metabolism. Toxicol. Mech. Methods.

[10] Fakhri Y., Mohseni-Bandpei A., Conti G.O., Ferrante M., Cristaldi A., Jeihooni A.K., Dehkordi M.K., Alinejad A., Rasoulzadeh H., Mohseni S.M. (2018). Systematic review and health risk assessment of arsenic and lead in the fished shrimps from the Persian gulf. Food Chem. Toxicol..

[11] Nagel A., Cuss C.W., Goss G.G., Shotyk W., Glover C.N. (2023). Accumulation of Thallium in Rainbow Trout (*Oncorhynchus mykiss*) Following Acute and Subchronic Waterborne Exposure. Environ. Toxicol. Chem..

[12] Pandiyan J., Mahboob S., Govindarajan M., Al-Ghanim K.A., Ahmed Z., Al-Mulhm N., Jagadheesan R., Krishnappa K. (2021). An assessment of level of heavy metals pollution in the water, sediment and aquatic organisms: A perspective of tackling environmental threats for food security. Saudi J. Biol. Sci..

[13] Huang H., Li Y., Zheng X., Wang Z., Wang Z., Cheng X. (2022). Nutritional value and bioaccumulation of heavy metals in nine commercial fish species from Dachen Fishing Ground, East China Sea. Sci. Rep..

[14] Wang J., Wang S.Y. (2012). Study on the present situation and causes of river health in Zhejiang Province. Econ. Res. Guide.

[15] Shorna S., Shawkat S., Hossain A., Quraishi S.B., Ullah A.K.M.A., Hosen M.M., Hossain K., Saha B., Paul B., Mamun H.A. (2021). Accumulation of Trace Metals in Indigenous Fish Species from the Old Brahmaputra River in Bangladesh and Human Health Risk Implications. Biol. Trace Elem. Res..

[16] (2012). National Standard for Food Safety Limits of Contaminants in Food.

[17] Ai L., Ma B., Shao S., Zhang L., Zhang L. (2022). Heavy metals in Chinese freshwater fish: Levels, regional distribution, sources and health risk assessment. Sci. Total Environ..

[18] Kalantzi I., Black K.D., Pergantis S.A., Shimmield T.M., Papageorgiou N., Sevastou K., Karakassis I. (2013). Metals and other elements in tissues of wild fish from fish farms and comparison with farmed species in sites with oxic and anoxic sediments. Food Chem..

[19] Sheikhzadeh H., Hamidian A.H. (2021). Bioaccumulation of heavy metals in fish species of Iran: A review. Environ. Geochem. Health.

[20] Castaldo G., Pillet M., Slootmaekers B., Bervoets L., Town R.M., Blust R., De Boeck G.J.A.T. (2020). Investigating the effects of a sub-lethal metal mixture of Cu, Zn and Cd on bioaccumulation and ionoregulation in common carp, *Cyprinus carpio*. Aquat. Toxicol..

[21] Gestin O., Lopes C., Delorme N., Garnero L., Geffard O., Lacoue-Labarthe T. (2022). Organ-specific accumulation of cadmium and zinc in Gammarus fossarum exposed to environmentally relevant metal concentrations. Environ. Pollut..

[22] Habib S.S., Batool A.I., Rehman M., Naz S. (2023). Assessment and Bioaccumulation of Heavy Metals in Fish Feeds, Water, and Some Tissues of *Cyprinus carpio* Cultured in Different Environments (Biofloc Technology and Earthen Pond System). Biol. Trace Elem. Res..

[23] Can E., Yabanli M., Kehayias G., Aksu Ö., Kocabaş M., Demir V., Kayim M., Kutluyer F., Şeker S. (2012). Determination of Bioaccumulation of Heavy Metals and Selenium in Tissues of Brown Trout Salmo trutta macrostigma (Duméril, 1858) from Munzur Stream, Tunceli, Turkey. Bull. Environ. Contam. Toxicol..

[24] Coulibaly S., Atse B.C., Koffi K.M., Sylla S., Konan K.J., Kouassi N.J. (2012). Seasonal accumulations of some heavy metal in water, sediment and tissues of black-chinned tilapia Sarotherodon melanotheron from Biétri Bay in Ebrié Lagoon, Ivory Coast. Bull. Environ. Contam. Toxicol..

[25] Yousafzai A.M., Ullah F., Bari F., Raziq S., Riaz M., Khan K., Nishan U., Sthanadar I.A., Shaheen B., Shaheen M. (2017). Bioaccumulation of Some Heavy Metals: Analysis and Comparison of *Cyprinus carpio* and *Labeo rohita* from Sardaryab, Khyber Pakhtunkhwa. Biomed. Res. Int..

[26] Salami I.R., Rahmawati S., Sutarto R.I., Jaya P.M. (2008). Accumulation of heavy metals in freshwater fish in cage aquaculture at Cirata Reservoir, West Java, Indonesia. Ann. N. Y. Acad. Sci..

[27] Wang J., Xiao J., Zhang J., Chen H., Li D., Li L., Cao J., Xie L., Luo Y. (2020). Effects of dietary Cu and Zn on the accumulation, oxidative stress and the expressions of immune-related genes in the livers of Nile tilapia (*Oreochromis niloticus*). Fish Shellfish Immunol..

[28] Saidon N.B., Szabó R., Budai P., Lehel J. (2024). Trophic transfer and biomagnification potential of environmental contaminants (heavy metals) in aquatic ecosystems. Environ. Pollut..

[29] Suedel B.C., Boraczek J.A., Peddicord R.K., Clifford P.A., Dillon T.M., Ware G.W. (1994). Trophic Transfer and Biomagnification Potential of Contaminants in Aquatic Ecosystems. Reviews of Environmental Contamination and Toxicology.

[30] Qian Y., Cheng C., Feng H., Hong Z., Zhu Q., Kolenčík M., Chang X. (2020). Assessment of metal mobility in sediment, commercial fish accumulation and impact on human health risk in a large shallow plateau lake in southwest of China. Ecotoxicol. Environ. Saf..

[31] Leung H.M., Duzgoren-Aydin N.S., Au C.K., Krupanidhi S., Fung K.Y., Cheung K.C., Wong Y.K., Peng X.L., Ye Z.H., Yung K.K.L. (2017). Monitoring and assessment of heavy metal contamination in a constructed wetland in Shaoguan (Guangdong Province, China): Bioaccumulation of Pb, Zn, Cu and Cd in aquatic and terrestrial components. Environ. Sci. Pollut. Res. Int..

[32] Yi Y.J., Zhang S.H. (2012). Heavy metal (Cd, Cr, Cu, Hg, Pb, Zn) concentrations in seven fish species in relation to fish size and location along the Yangtze River. Environ. Sci. Pollut. Res. Int..

[33] Guo Z., Gao Y., Yuan X., Yuan M., Huang L., Wang S., Liu C., Duan C. (2023). Effects of Heavy Metals on Stomata in Plants: A Review. Int. J. Mol. Sci..

[34] Chen Y.-G., He X.-L., Huang J.-H., Luo R., Ge H.-Z., Wołowicz A., Wawrzkiewicz M., Gładysz-Płaska A., Li B., Yu Q.-X. (2021). Impacts of heavy metals and medicinal crops on ecological systems, environmental pollution, cultivation, and production processes in China. Ecotoxicol. Environ. Saf..

[35] Senze M., Kowalska-Goralska M., Czyz K. (2023). Emergent (branched bur-reed-*Sparganium erectum* L.) and submergent (river water-crowfoot-Ranunculus fluitans Wimm., 1841) aquatic plants as metal biosorbents under varying water pH conditions in laboratory conditions. Environ. Sci. Pollut. Res. Int..

[36] Sricoth T., Meeinkuirt W., Saengwilai P., Pichtel J., Taeprayoon P. (2018). Aquatic plants for phytostabilization of cadmium and zinc in hydroponic experiments. Environ. Sci. Pollut. Res. Int..

[37] Beals C., King H., Bailey G. (2023). The peroxidase response of *Alternanthera philoxeroides* (Alligator Weed) and Nasturtium officinale (Watercress) to heavy metal exposure. Environ. Sci. Pollut. Res. Int..

[38] Sudarshan P., Mahesh M.K., Ramachandra T.V. (2020). Dynamics of Metal Pollution in Sediment and Macrophytes of Varthur Lake, Bangalore. Bull. Environ. Contam. Toxicol..

[39] Serafini R., Arreghini S., Troiani H.E., de Iorio A. (2023). Copper, zinc, and chromium accumulation in aquatic macrophytes from a highly polluted river of Argentina. Environ. Sci. Pollut. Res. Int..

[40] Ntakiyiruta P., Briton B.G.H., Nsavyimana G., Adouby K., Nahimana D., Ntakimazi G., Reinert L. (2022). Optimization of the phytoremediation conditions of wastewater in post-treatment by Eichhornia crassipes and Pistia stratiotes: Kinetic model for pollutants removal. Environ. Technol..

[41] de Vasconcelos V.M., de Morais E.R.C., Faustino S.J.B., Hernandez M.C.R., Gaudêncio H.R.d.S.C., de Melo R.R., Junior A.P.B. (2021). Floating aquatic macrophytes for the treatment of aquaculture effluents. Environ. Sci. Pollut. Res. Int..

[42] Li J., Yu H., Luan Y. (2015). Meta-Analysis of the Copper, Zinc, and Cadmium Absorption Capacities of Aquatic Plants in Heavy Metal-Polluted Water. Int. J. Environ. Res. Public Health.

[43] Gongtai T., Dengxuan D., Xinghua D., Guojing X., Jinlu Z., Minglei Z., Chunsheng W., Ming L., Min L., Yamei M. (2014). Study on Enrichment and Removal Effects of Eichhornia crassipes and Polygonum cuspidatum on Combined Pollution of Heavy Metals in Water Body. J. Yangtze Univ..

[44] Wenbing Z., Liangfeng T., Dahui L., Hua Y., Min Z., Duanwei Z. (2005). Research progress of Eichhornia crassipes and its resource utilization. J. Huazhong Agric. Univ..

[45] Nawirska A. (2005). Binding of heavy metals to pomace fibers. Food Chem..

[46] Piekut A., Gut K., ćwieląg-Drabek M., Domagalska J., Marchwińska-Wyrwał E. (2019). The relationship between children’s non-nutrient exposure to cadmium, lead and zinc and the location of recreational areas—Based on the Upper Silesia region case (Poland). Chemosphere.

[47] Banfalvi G. (2006). Removal of insoluble heavy metal sulfides from water. Chemosphere.

[48] Arshad K., Aqeel M., Noman A., Nazir A., Mahmood A., Rizvi Z.F., Sarfraz W., Hyder S., Zaka S., Khalid N. (2023). Ecological health risk assessment of microplastics and heavy metals in sediments, water, hydrophytes (*Alternanthera philoxeroides*, *Typha latifolia*, and *Ipomoea carnea*), and fish (*Labeo rohita*) in Marala wetlands in Sialkot, Pakistan. Environ. Sci. Pollut. Res. Int..

[49] Scofield B.D., Torso K., Fields S.F., Chess D.W. (2021). Contaminant metal concentrations in three species of aquatic macrophytes from the Coeur d’Alene Lake basin, USA. Environ. Monit. Assess..

